# The Perception of Psychosocial Risks and Work-Related Stress in Relation to Job Insecurity and Gender Differences: A Cross-Sectional Study

**DOI:** 10.1155/2018/7649085

**Published:** 2018-12-19

**Authors:** Simone De Sio, Fabrizio Cedrone, Edoardo Trovato Battagliola, Giuseppe Buomprisco, Roberto Perri, Emilio Greco

**Affiliations:** ^1^Research Unit of Occupational Medicine, “Sapienza” University of Rome, Rome, Italy; ^2^Department of Science for Health Promotion and Mother to Child Care “G. D'Alessandro”, University of Palermo, Palermo, Italy; ^3^Department of Sense Organs, “Sapienza” University of Rome, Rome, Italy; ^4^Department of Neurology and Psychiatry, “Sapienza” University of Rome, Rome, Italy

## Abstract

**Introduction:**

The perception of psychosocial risks exposes workers to develop work-related stress. Recently the attention of scientific research has focused on a psychosocial risk already identified as “job insecurity” that regards the “overall concern about the continued existence of the job in the future” and that also depends on worker's perception, different for each gender.

**Aim of the Study:**

The aim of this cross sectional study is to show if job insecurity, in the form of temporary contracts, can influence the perception of psychosocial risks and therefore increase worker's vulnerability to work-related stress and how the magnitude of this effect differs between genders.

**Materials and Methods:**

338 administrative technical workers (113 males and 225 females) were administered a questionnaire, enquiring contract typology (permanent or temporary contracts), and the Health Safety Executive questionnaire to assess work-related stress. The Health Safety Executive Analysis Tool software was used to process collected questionnaires and the Wilcoxon rank-sum test was used to evaluate the statistical significance of the differences obtained.

**Results:**

Workers with temporary contracts obtained lower scores than workers with permanent contracts in all the domains explored by the Health Safety Executive Analysis questionnaire, statistically significant (P<0,05). The female workers obtained lower scores than male workers in all domains explored by the Health Safety Executive questionnaire.

**Conclusions:**

Authors conclude that perception of psychosocial risks can be influenced by job insecurity, in the form of temporary contracts, and increases worker's vulnerability to work-related stress and differs between genders.

## 1. Introduction

World Health Organization (WHO) defines work-related stress as “a condition characterized by physical, psychological, or social suffering or dysfunction, which arises from the feeling of not being able to respond to requests or to live up to expectations” [1]. Work-related stress (WRS) is a complex phenomenon and develops when multiple psychosocial risk factors coexist and interact. Psychosocial risks arise from the interaction from work content, work organization, technological and environmental conditions, and workers' skills, resources, and needs [2].

The psychosocial risks that influence more the perception of stress are excessive workloads, a lack of decisional autonomy in the management of one's work, a lack of support by colleagues or superiors, the presence of relational conflicts in the workplace, the under evaluation of one's role within the company, and a the lack of involvement in the changes of company organization [3]. Chronic exposure to psychosocial risks has been associated with a wide range of mental and physical disorders, including anxiety, depression, suicide attempts, sleep disorders, back pain, chronic fatigue, digestive problems, autoimmune diseases, impaired immune function, cardiovascular diseases, hypertension, and peptic ulcers [4–12].

In 2008, a deep economic crisis started in the US and rapidly spread around the world, severely affecting the labor market. In this context many companies had to reduce the number of workers to limit their expenses and reorganized their internal structure to maintain the same level of efficiency and competitiveness, also by using different types of work contract: permanent contracts, temporary contracts, agency contracts, freelancers, and zero hour contracts [13–15].

The subsequent increase in the precariousness of employment focused the attention of scientific researches on a psychosocial risk already identified as “job insecurity” [16].

Job insecurity is regarded as the “overall concern about the continued existence of the job in the future” [17]. One of the causes of job insecurity is temporary work contracts, because they do not guarantee to workers the prospective of future work [18–24].

Job insecurity not only depends on objective conditions, such as different contract typologies, but also depends on the worker's perception of their situation, which is different for each gender, as well as their cognitive evaluation, coping skills, and social support [25–27].

Gender has significant implications on the role that workers are likely to assume within the company. For example, female workers tend to be less influential on social dynamics than their male counterparts are, and this phenomenon often leads to less prestigious roles, lower salaries [28], lower overall job satisfaction [29], and consequently lower performance at work [30].

The aim of this research is to show, through a cross sectional study, if the perception of psychosocial risks can depend on job insecurity, in the form of temporary contracts and can increase worker's vulnerability to work-related stress and how the magnitude of this effect differs between genders.

## 2. Methods

During the health surveillance activities carried out pursuant to the current legal framework in 2017, the authors included in this cross-sectional study a population of *N* = 338 administrative technical workers (113 males and 225 females) employed at the same company, with an 8:30 a.m.–17:15 p.m. working time.

A clinical medical history questionnaire was administered to all subjects, with details about contract typology (permanent or temporary contracts); contract typology was corporate's exclusive decision and was not agreed with workers. Temporary contracts are biennial in length and can be either renewed or converted into permanent contracts on their expiration.

The Health and Safety Executive (HSE) questionnaire was also administered to all subjects for WRS assessment.  Table 1 shows the structure of the sample.

The HSE questionnaire was developed by the Health and Safety Executive [31, 32].

The questionnaire is a useful tool designed to assess working conditions likely to cause work-related stress; it consists of 35 items rated on 5-point Likert scale, where higher scores indicate better working conditions and lower stress risk and define 7 different domains corresponding to as many primary factors of work-related stress risk:

- Demands: it explores issues such as workload, work patterns, and the working environment.
- Control: it focuses on workers' decision-making autonomy.
- Support: this domain is analyzed and divided into two types, namely, in terms of “support from managers” and “support among colleagues”, and includes encouragement, sponsorship, and resources provided by the organization, line management, and colleagues.
- Relationships: it explores promotion of positive work practices to avoid conflicts and deal with unacceptable behaviour.
- Role: whether workers understand their role within the organization and whether the organization ensures that, no conflicts occur.
- Change: how organizational change (large or small) is managed and communicated within the organization.

Questionnaires were uploaded to the HSE Analysis Tool, a specific software that analyzes them and classifies workers into four risk groups for each of the seven domains:

(i) Those below the 20th percentile (20% of the lowest reference values), for which corrective action is urgently required (d).

(ii) Those below average (<50%, but still above the 20th percentile rank), for which corrective action is required (c).

(iii) Those at or above average (≤50%), but below the 80th percentile and not requiring action (b).

(iv) Those at or above the 80th percentile, for which no corrective action is required (a).

Comparison with the benchmark was used to establish priorities for action and to set short- and long-term performance targets for each of the scales [25].

The HSE Analysis Tool software was used to process collected questionnaires and three distinct profiles (total population, male population, and female population) were highlighted, in relation to contract typology (permanent and temporary contract).

Subsequently, authors used a nonparametric statistical analysis for independent samples, Wilcoxon rank-sum test, to evaluate the statistical significance of the differences in scores obtained for each of the seven domains of the three distinct profiles (total population, male population and female population), in relation to contract typology (permanent and temporary contract).

All questionnaires were self-administered, collected, and checked to make sure they had been properly and fully completed. All subjects confirmed their awareness of the sensitive nature of the data being collected and agreed for this data to be processed anonymously and collectively, through the appropriate scientific procedures, in accordance with the principles of the Declaration of Helsinki.

The statistical calculations were performed using STATA 14 software.

## 3. Results

### 3.1. Total Population (Males and Females)

As shown in Table 2, the total population with temporary contracts reported low HSE scores (c-d) in all the explored domains; instead the total population with permanent contracts reported low HSE scores (c-d) in three domains: Managers support, Peer support, and Relationship. The Wilcoxon rank-sum test showed significant differences (p value < 0,05) in all explored domains, between the population with temporary contracts and the population with permanent contracts.

### 3.2. Male Population

As shown in Table 3, the male population with temporary contracts reported low HSE scores (c-d) in the domains: Control, Managers support, Peer support, Relationships, and Role; instead male workers with permanent contracts reported low HSE scores (c-d) in the Relationship domain. The Wilcoxon rank- showed significant differences (p value < 0,05) in all explored domains, except for the Demand domain, between the male population with temporary contracts and the male population with permanent contracts.

### 3.3. Female Population

As shown in Table 4, the female population with temporary contracts reported low HSE scores (c-d) in all the explored domains; instead female workers with permanent contracts reported low HSE scores (c-d) in domains: Managers support, Peer support, Relationships, and Role. The Wilcoxon rank-sum test showed significant differences (p value < 0,05) in domains: Demand, Control, Relationship, and Role, between the female population with temporary contracts and the female population with permanent contracts.

## 4. Conclusion 

Our analysis showed that workers with temporary contracts, compared to workers with permanent contract, are more vulnerable to psychosocial risks, which increase susceptibility to develop WRS. In fact, the results obtained showed that the total population with temporary contracts requires corrective interventions in all domains, whereas workers with permanent contracts require corrective interventions on three domains: Manager support, Peer support, and Relationship. As already described in literature, contract typology (in the form of either temporary or permanent contracts) can impact worker's wellness and corporate stability in two ways: by worsening social relations with both peers and superiors and by lowering performance level [33]. In fact, workers with temporary contracts report less overall satisfaction and lower wellness levels, as a direct effect of the uncertainty of their future employment [34].

Our analysis also showed that female workers, compared to male workers, are more vulnerable to psychosocial risks, regardless of contract typology, and this increases their susceptibility to develop WRS.

In fact, the results obtained showed that female workers with temporary contracts require corrective interventions in all domains, whereas male workers with temporary contracts require corrective interventions in the domains: Control, Managers support, Peer support, Relationships, and Role.

Female workers with permanent contracts require corrective interventions in the domains: Managers support, Peer support, Relationships, and Role, whereas male workers with permanent contracts require corrective interventions in the domain Relationship.

This finding emphasizes that female workers are more vulnerable to psychosocial risks and that gender differences must be considered for WRS assessment and prevention [35].

Some studies have already demonstrated that the perception of psychosocial risks and WRS depends on cognitive evaluation and coping strategies, which differ between genders [36]. For example, male workers tend to adopt problem-focused coping behaviours, which are effective in dealing with emerging problems; instead, female workers are more likely to adopt emotional-focused coping behaviours, which lead to more introspection and make them more vulnerable to the effects of stress on mental health [37, 38]. In addition, the greater vulnerability that female workers showed might be related to social norms, exposing the female gender more widely to work-family conflicts [39]. This topic has gained so much relevance that the European Occupational Safety and Health Agency (OSHA-EU) has presented the need for additional research on work-family interactions, with the aim of identifying the balance most compatible with health [40].

Job insecurity, in the form of temporary contracts, influences the perception of psychosocial risks and increases worker's vulnerability to WRS, even after adjusting for gender. As a result, the combination of job insecurity, in the form of temporary contracts, and female gender increases worker's susceptibility to WRS.

## Figures and Tables

**Table 1:** Population data: mean ages (standard deviation), contract type.

	**Num (**%**)**	**Mean Age (SD)**	**Contract type**	
			**Permanent contract** **N (**%**)**	**Temporary contract** **N (**%**)**
**Total **	338 (100%)	44,14 (12,5)	206 (61%)	132 (39%)
**Males **	113 (33,43%)	44,33 (11,8)	68 (60%)	45 (40%)
**Females**	225 (66,57%)	44,03 (12,9)	138 (61%)	87 (39%)

**Table 2:** Total population (male and female): HSE score of permanent and temporary workers, median, first (Q1), and third (Q3) quartiles of the HSE scores obtained and statistical significance by Wilcoxon rank-sum test.

	**Permanent contract N=206**		**Temporary contract N=132**		***P*-value**
	**HSE score**	**Median value** **(Q1-Q3)**	**HSE score**	**Median value** **(Q1-Q3)**	
**Demands **	3,25b	3,25 (2,87-3,62)	2,98c	2.875 (2,50-3,37)	p=0.000
**Control **	3,64b	3,66 (3,16-4,16)	3,23c	3,16 (2,83-3,66)	p=0.000
**Managers' Support **	3,35c	3,40 (2,80-4,00)	3,06d	3,10 (2,40-3,70)	p=0.002
**Peer Support **	3,66c	3,75 (3,25-4,00)	3,48d	3,50 (3,00-4,00)	p=0.034
**Relationships **	3,46d	3,50 (2,75-4,25)	3,07d	3,00 (2,25-4,00)	p=0.000
**Role **	4,21b	4,20 (4,00-4,80)	3,74d	3,80 (3,40-4,20)	p=0.000
**Change **	3,24a	3,33 (2,66-4,00)	2,96c	3,00 (2,16-3,66)	p=0.003

^a^Performance classified as very good. ^b^Performance classified as good, with potential for improvement. ^c^Performance classified as requiring improvement. ^d^Performance classified as requiring urgent improvement measures.

**Table 3:** Male population: HSE score of permanent and temporary workers, median, first (Q1), and third (Q3) quartiles of the HSE scores obtained and statistical significance by Wilcoxon rank-sum test.

	**Permanent contract N=68**		**Temporary contract N=45**		***P*-value**
	**HSE score**	**Median value** **(Q1-Q3)**	**HSE score**	**Median value** **(Q1-Q3)**	
**Demands **	3,22b	3,25 (2,75-3,62)	3,11b	3,00 (2,75-3,50)	p=0.186
**Control **	3,96a	4,00 (3,33-4,66)	3,28c	3,33 (2,83-3,83)	p=0.000
**Managers' Support **	3,53b	3,40 (2,80-4,20)	3,12d	3,20 (2,60-3,60)	p=0.009
**Peer Support **	3,78b	3,75 (3,25-4,37)	3,39d	3,25 (3,00-3,75)	p=0.008
**Relationships **	3,51d	3,75 (2,75-4,50)	3,02d	2,75 (2,50-3,50)	p=0.007
**Role **	4,40a	4,60 (4,00-4,80)	3,58d	3,60 (3,00-4,20)	p=0.000
**Change **	3,63a	3,66 (3,16-4,00)	3,14b	3,00 (2,66-3,66)	p=0.000

^a^Performance classified as very good. ^b^Performance classified as good, with potential for improvement. ^c^Performance classified as requiring improvement. ^d^Performance classified as requiring urgent improvement measures.

**Table 4:** Female population: HSE score of permanent and temporary workers, median, first (Q1), and third (Q3) quartiles of the HSE scores obtained and statistical significance by Wilcoxon rank-sum test.

	**Permanent contract N=138**		**Temporary contract N=87**		***P*-value**
	**HSE score**	**Median value** **(Q1-Q3)**	**HSE score**	**Median value** **(Q1-Q3)**	
**Demands **	3,28b	3,25 (2,87-3,62)	2,93d	2,87 (2,37-3,37)	p=0.000
**Control **	3,49b	3,50 (3,00-4,00)	3,22d	3,16 (2,83-3,50)	p=0.000
**Managers' Support **	3,29c	3,40 (2,60-4,00)	3,05d	3,00 (2,40-3,80)	p=0.050
**Peer Support **	3,61d	3,70 (3,25-4,00)	3,55d	3,50 (3,00-4,00)	p=0.508
**Relationships **	3,46d	3,50 (2,75-4,25)	3,12d	3,25 (2,25-4,00)	p=0.035
**Role **	4,13c	4,20 (3,80-4,60)	3,84d	3,80 (3,40-4,40)	p=0.000
**Change **	3,08b	3,00 (2,66-3,66)	2,89c	3,00 (2,00-3,66)	p=0.127

^a^Performance classified as very good. ^b^Performance classified as good, with potential for improvement. ^c^Performance classified as requiring improvement. ^d^Performance classified as requiring urgent improvement measures.

## Data Availability

The data used to support the findings of this study are included within the article.
