# Corrigendum to “The Perception of Psychosocial Risks and Work-Related Stress in Relation to Job Insecurity and Gender Differences: A Cross-Sectional Study”

**DOI:** 10.1155/2019/3209787

**Published:** 2019-11-21

**Authors:** Simone De Sio, Fabrizio Cedrone, Edoardo Trovato Battagliola, Giuseppe Buomprisco, Roberto Perri, Emilio Greco

**Affiliations:** ^1^Research Unit of Occupational Medicine, “Sapienza” University of Rome, Rome, Italy; ^2^Department of Science for Health Promotion and Mother to Child Care “G. D'Alessandro”, University of Palermo, Palermo, Italy; ^3^Department of Sense Organs, “Sapienza” University of Rome, Rome, Italy; ^4^Department of Neurology and Psychiatry, “Sapienza” University of Rome, Rome, Italy

In the article titled “The Perception of Psychosocial Risks and Work-Related Stress in Relation to Job Insecurity and Gender Differences: A Cross-Sectional Study”, reference [13] should be removed, since it is not appropriately cited. The cited article is focused on the correlation between stress and economic crisis, whereas the citation is added when talking about companies' reaction to the economic crisis. Accordingly, the third paragraph in Introduction should be corrected as follows.

“In 2008, a deep economic crisis started in the US and rapidly spread around the world, severely affecting the labor market. In this context, many companies had to reduce the number of workers to limit their expenses and reorganized their internal structure to maintain the same level of efficiency and competitiveness, also by using different types of work contract: permanent contracts, temporary contracts, agency contracts, freelancers, and zero-hour contracts [15].”
